# Podcast about child care as an educational health technology: a qualitative study

**DOI:** 10.15649/cuidarte.3845

**Published:** 2024-09-01

**Authors:** Joyce de Oliveira Borges, Fernanda Garcia Bezerra Góes, Aline Cerqueira Santos Santana da Silva, Fernanda Maria Vieira Pereira Ávila, Maithê de Carvalho Lemos e Goulart, Nátale Gabriele Ferreira Nunes, Vanessa Ramos Martins

**Affiliations:** 1 Fluminense Federal University, Rio de Janeiro, Brazil. E-mail: joyce borges@id.uff.br Fluminense Federal University Rio de Janeiro Brazil joyce borges@id.uff.br; 2 Fluminense Federal University, Rio de Janeiro, Brazil. E-mail: ferbezerra@gmail.com Fluminense Federal University Rio de Janeiro Brazil ferbezerra@gmail.com; 3 Fluminense Federal University, Rio de Janeiro, Brazil. E-mail: alinecer2014@gmail.com Fluminense Federal University Rio de Janeiro Brazil alinecer2014@gmail.com; 4 Fluminense Federal University, Rio de Janeiro, Brazil. E-mail: fernandamvp@id.uff.br Fluminense Federal University Rio de Janeiro Brazil fernandamvp@id.uff.br; 5 Fluminense Federal University, Rio de Janeiro, Brazil. E-mail: maithegoulart@gmail.com Fluminense Federal University Rio de Janeiro Brazil maithegoulart@gmail.com; 6 Fluminense Federal University, Rio de Janeiro, Brazil. E-mail: natalenunes@id.uff.br Fluminense Federal University Rio de Janeiro Brazil natalenunes@id.uff.br; 7 Federal University of the State of Rio de Janeiro, Rio de Janeiro, Brazil. E-mail: vrmartins@edu.unirio.br Federal University of the State of Rio de Janeiro Rio de Janeiro Brazil vrmartins@edu.unirio.br

**Keywords:** Family, Child, Educational Technology, Health Education, Podcast, Familia, Niño, Tecnología Educacional, Educación en Salud, Podcast, Família, Criança, Tecnologia Educacional, Educação em Saúde, Podcast

## Abstract

**Introduction:**

Technological advances have transformed communication, making social media essential elements that promote easy access to information.

**Objective:**

To understand the use of podcasts as an educational health technology for disseminating knowledge about child care.

**Materials and Methods:**

Online, descriptive, and exploratory study, with a qualitative approach, developed between March and April 2023, using an electronic form with 41 participants, listeners of the podcasts from a technological initiation project. The data was processed in the Interface de R pour les Analyses Multidimensionnelles de Textes et de Questionnaires and analyzed according to Thematic Content Analysis.

**Results:**

The use of the podcast was described as fundamental in the face of different doubts about child care. This educational technology proved enlightening, practical, accessible, and interesting, and the topics addressed were important for expanding the listeners’ knowledge. Furthermore, the fact of having an audio format made consuming the content and daily tasks easier.

**Discussion:**

Changes in the participants’ behavior were observed through the use ofthis social media, encouraging safe practices and guidance that corroborate health education practices. It has reinforced the importance of social media as a means of disseminating information to improve care practices aimed at the pediatric population.

**Conclusions:**

The podcast is an educational health technology that eases the dissemination of knowledge to the population about child care in a free, practical, and accessible way and can, therefore, be applied to health education, having an innovative character from the perspective of digital health.

## Resumen.

**Introducción:**

Los avances tecnológicos han transformado la comunicación, convirtiendo a las redes sociales en elementos esenciales que facilitan el acceso a la información.

**Objetivo:**

Comprender el uso de podcasts como tecnología educativa en salud para difundir conocimientos sobre el cuidado de la infancia.

**Materiales y Métodos:**

Estudio online, descriptivo y exploratorio, con enfoque cualitativo, desarrollado entre marzo y abril de 2023, mediante formulario electrónico con 41 participantes, oyentes de podcasts de un proyecto de iniciación tecnológica. Los datos fueron procesados en Interface de R pour Analyses Multidimensionnelles de Textes et de Questionnaires y analizados según el Análisis de Contenido Temático.

**Resultados:**

El uso del podcast fue calificado como fundamental ante diferentes dudas sobre el cuidado de la infancia. Esta tecnología educativa resultó esclarecedora, práctica, accesible e interesante; los temas tratados fueron importantes para ampliar los conocimientos de los oyentes. Además, tener un formato de audio facilitó el consumo del contenido junto con las tareas diarias.

**Discusión:**

Se observaron cambios en el comportamiento de los participantes a través del uso de esta red social, incentivando prácticas seguras y orientaciones que corroboran las prácticas de educación en salud. Fortaleció la importancia de las redes sociales como medio de difusión de información para mejorar las prácticas de atención dirigidas a la población pediátrica.

**Conclusiones:**

El podcast es una tecnología educativa en salud que facilita la difusión de conocimientos a la población sobre el cuidado infantil de forma práctica, accesible y gratuita y puede, por tanto, ser aplicado a la educación para la salud, teniendo un carácter innovador desde la perspectiva de la salud digital.

## Resumo.

**Introdução:**

Os avanços tecnológicos têm transformado a comunicação, tornando as mídias sociais elementos essenciais que facilitam o acesso à informação.

**Objetivo:**

Compreender o uso do podcast como tecnologia educacional em saúde para a disseminação de conhecimentos sobre cuidados na infância.

**Materiais e Métodos:**

Estudo online, descritivo e exploratório, de abordagem qualitativa, desenvolvido entre março e abril de 2023, por meio de formulário eletrônico com 41 participantes, ouvintes dos podcasts de um projeto de iniciação tecnológica. Os dados foram processados no Interface de R pour les Analyses Multidimensionnelles de Textes et de Questionnaires e analisados segundo Análise de Conteúdo Temática.

**Resultados:**

O uso do podcast foi descrito como fundamental frente às distintas dúvidas sobre os cuidados na infância. Essa tecnologia educacional revelou-se esclarecedora, prática, acessível e interessante, cujos temas abordados foram importantes para a ampliação do conhecimento dos ouvintes. Ademais, por possuir formato de áudio facilitou o consumo do conteúdo juntamente com as tarefas diárias.

**Discussão:**

Observaram-se mudanças no comportamento dos participantes através do uso desta mídia social, estimulando práticas e orientações seguras que corroboram com as práticas de educação em saúde. Fortaleceu a importância das mídias sociais como um meio de divulgação de informações para melhoria de práticas de cuidado voltadas à população pediátrica.

**Conclusões:**

O podcast é uma tecnologia educacional em saúde facilitadora na disseminação de conhecimentos para a população sobre os cuidados infantis de forma prática, acessível e gratuita podendo, portanto, ser aplicada para a educação em saúde, possuindo um caráter inovador na perspectiva da saúde digital.

## Introduction

Scientific and technological advances, driven by information and communication technologies (ICT) and the internet, have promoted accelerated human interactions. The advent ofsmartphones, operating as real computers accessible to most of the global population on a full-time basis, has significantly impacted behavior patterns. This transformation influences the dynamics of societies, including new paradigms in the health-disease process. In the context of ICT, innovative strategies are emerging to disseminate knowledge, providing better conditions to ensure the right to health^1^.

The term “social media” is often mistakenly used as a synonym for “social network.” However, a “social network” is a group of people who are connected in some way with the aim of interacting with each other, virtually or otherwise. Social media are digital platforms like Facebook, YouTube, Twitter, Instagram, and Spotify^2^. Nowadays, online interaction between individuals has become vital for disseminating information, highlighting social media as essential tools for health education in the digital age^3^.

Social media are part of people’s lives and are important communication channels that actively contribute to daily human actions^4^. They offer effective channels for disseminating safe guidance, promoting health, and combating fake news, reaching a wider audience^5^. The development of educational materials that disseminate truthful information has a positive impact on individuals’ self care process^5^^, ^^6^. Nevertheless, the sharing of technical-scientific knowledge by health professionals must take place in a simple, comprehensible, and attractive way, aiming for a better understanding of the population^7^.

Health technologies are categorized as managerial, care-related, and educational^8^. Among educational technologies, podcasts stand out as a social media platform in audio format and a recent ICT (the first Brazilian production was in 2005), compared to traditional programs like radio and television. The podcast is innovative, combining the most modern and traditional elements of education, the online environment and orality^9^. Its flexibility of reproduction and sharing allows users to consume content at the time and place of their choice, facilitating the dissemination of knowledge beyond geographical barriers^10^.

The use of podcasts contributes to the expansion of digital health, as set out in the Global Digital Health Strategy (2020-2025), which has gained increased relevance during the pandemic. Digital health aims to improve health for everyone, everywhere, by accelerating the development and adoption of person-centered, appropriate, accessible, affordable, scalable, and sustainable digital health solutions to prevent, detect, and treat health problems to achieve the health-related sustainable goals^11^. Accordingly, in 2021, a technological initiation project was created, linked to scientific research and university extension, to share child care guidance with the public across different social media platforms, including publishing podcast episodes on an audio streaming platform.

However, in order to assess the effectiveness of this educational health technology in disseminating knowledge about child care, it was essential to understand the listeners’ perspectives. Furthermore, when searching the literature about the use of podcasts with families in the context of child care, no results were found, which is why this study is justified. The aim was, therefore, to understand the use of podcasts as an educational health technology for disseminating knowledge about child care.

## Materials and Methods

An online, descriptive, and exploratory study with a qualitative approach, following the Consolidated Criteria for Reporting Qualitative Research (COREQ) was developed in a virtual environment between March and April 2023. This enabled the participation of individuals from any geographic location. The dataset was stored in Mendeley Data^12^.

The population consisted of listeners to the podcasts of a technological initiation project focused on disseminating convergent, truthful and quality transmedia content to families of newborns and children. The project began its activities in August 2021 with a team dedicated to sharing reliable guidance to families, in order to promote safe and qualified child care, especially during the pandemic. Among the topics covered, can be highlighted: wearing masks, using 70% alcohol, returning to school, newborn stimulation, mental health, vaccination, breastfeeding, child violence, immune system and nutrition, childhood bereavement, and visiting newborns. The podcasts are available for free on Spotify®.

The inclusion criteria were podcast listeners who were parents, guardians, relatives, health professionals and/or students aged over 18. The exclusion criteria were podcast listeners who had physical and/or mental limitations preventing them from answering the online form and/or who were illiterate. Theoretical saturation was used for sample delimitation due to the recurrence of words and meanings^13^. As a result, 41 listeners participated in the project.

An online form was created using Google Forms. The first part contained closed questions to characterize the participants. With regard to the study’s object, ten open-ended questions were included covering topics such as listening frequency, listening experience, influence on child care, dissemination of knowledge, practical application of the content, changes in practices, perceived benefits and difficulties, sharing with acquaintances, as well as comments and suggestions about the podcasts.

For data collection, the participants were selected for convenience by inviting them via the project’s social media (Facebook, Instagram and WhatsApp) and posting a link with information about the nature and confidentiality of the study data. After accepting the Free and Informed Consent Form, participants were directed to fill in the form. It took 15 minutes to complete the questionnaire and there were no withdrawals.

For the analysis of the characterization data, Microsoft Office Excel® was used, applying descriptive statistics with measures of absolute and relative frequencies, as well as measures of central tendency. The qualitative data analysis was conducted in three stages: 1) preparing and coding the textual corpus; 2) processing the textual data in the software, and 3) interpreting the findings. The discursive answers extracted from the forms constituted the primary data source for analysis using the Interface de R pour les Analyses Multidimensionnelles de Textes et de Questionnaires (IRAMUTEQ) software. The following analytical methods were used: classical textual statistics, word cloud, similarity analysis, and descending hierarchical classification (DHC). Active forms with a *x2* value of 3.84 or higher (p<0.05) were selected to determine the strength of word association within the class, especially those with p<0.0001 indicating a very strong association^14^.

After the textual processing, the interpretative process was conducted based on the assumptions of the Thematic Content Analysis (TCA) ^15^. Through reading the text segments, data inference and interpretation were carried out based on the core meaning of the answers.

The study was approved by the Research Ethics Committee (CAAE: 66077322.2.0000.5243, Opinion: 5.944.449). The participants were informed about the study and provided their consent to participate. To ensure anonymity, an alphanumeric code was used according to the order of participation.

## Results

A total of 41 (100.00%) people participated in the research, where 34 (82.93%) were relatives or guardians of children, of whom 15 (36.59%) were mothers, 8 (19.51%) were other relatives, 5 (12.20%) were uncles, 5 (12.20%) were cousins, 4 (9.76%) were grandfathers, 3 (7.32%) were fathers and 1 (2.44%) was brother. The average age was 31.6 years, ranging from 21 to 60 years; 34 (82.93%) were female. In terms of education, 23 (56.10%) had completed higher education, 11 (26.83%) had completed high school, 2 (4.88%) had a graduate degree, 4 (9.76%) had not completed higher education and 1 (2.44%) had completed elementary school. Regarding occupation, 15 (36.59%) were health students, 10 (24.39%) health professionals, 8 (19.51%) students, 3 (7.32%) teachers, 3 (7.32%) nurses, 2 (4.88%) homemakers, and 25 (60.98%) were distributed in other professions. Most participants lived in the Southeast region of Brazil (31 people, (75.61%) with others from the South (5 people, 12.20%), Northeast (4 people, 9.76%), and Central-West (1 person, 2.44%).

**Figure 1:**
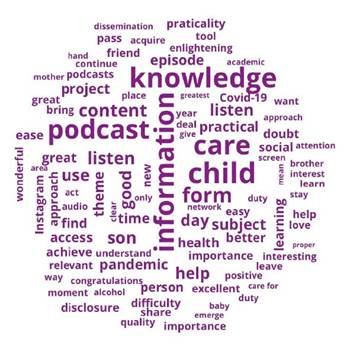
Word Cloud

According to Classical Textual Statistics, the textual corpus comprised 41 texts with a total of 4,608- word occurrences, with 871 being distinct words. Of these, 443 (9.61%) were single occurrences (hapax), with an average occurrence of 112.39 per text. In order to create the Word Cloud shown in Figure 1, words that appeared at least five times in the textual corpus and the most recurrent were included, removing the adverbs, namely: “information,” “podcast,” “child,” “knowledge” and “form,” which appear respectively 42, 37, 37, 34 and 30 times in the textual corpus.

In the interpretative process, it was understood that the active forms “information,” “knowledge,” “child” and “form” are central and strongly interlinked within the textual corpus. The podcast is recognized as an educational health technology that contributes to the dissemination of information and knowledge to the audience in a simple and easy-to-understand way, aiding in the correct care of children. The podcast was described as fundamental, especially during the pandemic, when there were many uncertainties about the care that guardians could offer to children.

*“It has helped us to deal with children in the family and brought more knowledge, to learn things in a simple way.” (P30) “It’s a very good exchange with the guests, and the information is really conveyed in the best way possible.” (P20) “The podcast was fundamental in this scenario of uncertainty that we were experiencing, having quality information that taught us how to care for children correctly.” (P19)*

**Figure 2:**
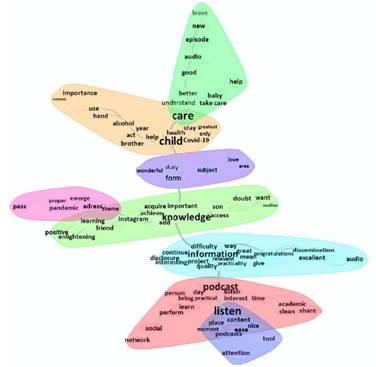
Similarity Analysis

The Similarity Analysis also included the active forms that recurred five times or more. This analysis made it possible to identify the distribution of the textual corpus in eight distinct clusters, and the five words with the highest recurrence were connected in a central and thicker line, reinforcing the strong correlation between these words. From these most relevant terms, several branches were generated with other words that also connected with these words within the clusters, as displayed in Figure 2.

The strong connection of the active forms “listening,” “podcast,” “information,” “knowledge,” “theme,” “form,” “child” and “care” reinforced the relevance of using this educational technology in audio format as a way to disseminate knowledge and information about child care. In the red cluster, the linkage of the term “podcast” with the words “practical,” “learn,” “interest” and “share” ratified the use of podcasts as an educational health technology, considering its practicality in the teaching-learning process by sharing knowledge about different topics in a simple way. In the dark blue cluster, which intersects with the red cluster, the word “listen” was connected with different terms, including “content,” “nice,” “ease,” “moment” and “place”, which revealed advantages of the use of this technology, as this digital media can be accessed at any time and place that the listener wishes.

The words “knowledge” and “information” assumed a certain position of centrality in the graphic structure, confirming that the podcast episodes contributed to the expansion of knowledge about childhood care. In the blue cluster, the word “information” branched out to the words “relevant,” “interesting,” “practicality,” “disclosure,” “dissemination” and “audio”, highlighting that the information disseminated by the episodes in audio format is interesting and promotes practicality for those who access them. In addition, branching from the term “knowledge”, within the green cluster, the words “acquire,” “easy,” “important,” “aggregate,” “learning,” “enlightening” and “child” invigorated the importance of this educational health technology, as it is easy to use, enabling learning and clarification of doubts about child care.

In the orange cluster, which intersects with the light green cluster, the word “child” has a strong linkage with the terms “help,” “COVID-19,” “health,” “deal,” “alcohol” and “hand”, which demonstrates that the podcast was an important tool to help parents to deal with the challenges of caring for children, especially during the COVID-19 pandemic. In this cluster, the words “alcohol,” “hand” and “use” stand out, referring to the topic of one of the podcast episodes (use of 70% alcohol during childhood) that was recurrently mentioned in the textual segments.

The light green, purple and pink clusters highlighted the words “care,” “form” and “theme” in this order. The word “care” branched out to some terms, including “understand,” “good,” “episode,” “care for,” “better,” “new” and “leave”, showing that the podcast episodes were a way to better understand the necessary care and that the participants were waiting for the release of new episodes. The purple cluster highlighted the word “form” that connected with the terms “wonderful,” “duty,” “theme,” “love” and “area”, indicating how satisfied listeners were with the project for sharing pertinent topics about child care. Furthermore, when branching from the term “theme” within the pink cluster, the words “address,” “pandemic,” “emerge” and “pass” showed that the topics covered in the podcast were important to those who have lived through the pandemic.

*“The possibility of listening to interesting content, with reliable sources of information, and the ease of listening to this information while carrying out daily activities. I listen to podcasts while washing dishes, sweeping the house or driving.” (P01)*

*“Enlightenment in an objective and clear way.” (P05)*

*“Being more careful with our children and babies.” (P06)*

*“The world today is linked to the digital universe, where every day more people turn to social media to learn or ask questions.” (P11)*

*“Use of alcohol on the hands. I was unsure whether it was correct to use it before the age of two and I heard about the importance of washing my hands with soap and water.” (P13)*

*“Access to quality information free of charge, easy to disclose. Adherence to applicable scientific knowledge, in accessible language.” (P14)*

*“Whenever there are new episodes, I’m listening.” (P16)*

**Figure 3:**
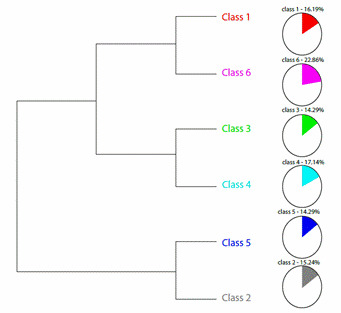
Descending Hierarchical Classification Dendrogram

In the Reinert method, a dendrogram was generated in the DHC, in which 136 text segments were found, with classification of 105 of them, that is, a success rate of 77.21%, forming six textual classes. As displayed in Figure 3, the class with the highest percentage was class 6 (pink), corresponding to 22.86% of text segments; followed by class 4 (blue), with 17.14%, and class 1 (red), with 16.19%, followed by class 2 (gray), with 15.24%, and, finally, classes 5 (blue) and 3 (green), with 15.24% each.

**Figure 4:**
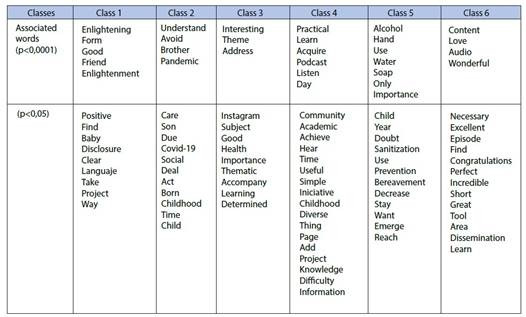
Distribution of active forms with p<0.0001 and p<0.05 by classes

The textual corpus was divided into two independent blocks (subcorpus). The first block consists of two subdivisions, the first one includes the classes 1 and 6, and the second one includes the classes 3 and 4, showing a greater proximity and homogeneity between them, since they have closer semantic content, but with a certain differentiation. The second block presents classes 2 and 5, which shows that they are more isolated from the others, with their semantic content being more distant. The analytical process culminated in the naming of two class grouping blocks, based on semantic content, especially the most relevant words (Figure 4).

### Block 1 - The use of podcasts in the dissemination of relevant content on child care in an enlightening, practical, accessible and interesting way

In class 1, the most relevant terms revealed the contribution of the podcast episodes to enlightening about child care, given the disclosure of easily accessible scientific knowledge and accessible language. The research participants described the contribution ofthis podcast as enlightening, accessible, clear and objective.

*“The way it was developed was very enlightening, since there were professionals in each area to talk about the theme.” (P29)*

*“Of great value, as it informs in a clear and objective way, not taking too long and arousing the interest of those listening.” (P05)*

*“The experience was pretty good, and the language is very accessible.” (P09)*

The participants also highlighted the importance of podcasts today as communication tools for disseminating information about child care, describing them as relevant and positive.

*“The fact that you can listen and learn through this podcast is a great communication tool.” (P26)*

*“Very positive. Very relevant, especially for people with restricted access to child development.” (P28)*

In class 6, it was observed that the podcast is a tool that enables the dissemination of useful content so that knowledge could be put into practice in an effective way.

*“Nice tool for disseminating knowledge.” (P27)*

*“Dissemination of useful, practical and important knowledge for those who work and coexist with the childhood universe.” (P35)*

In this class, the semantic repertoire was composed of many words praising the project’s podcast, such as “excellent,” “wonderful,” “perfect,” “incredible” and “nice”. These words emerged when the participants described their experiences in listening to the episodes.

*“Podcasting is on the rise. It’s an excellent way to reach people (P14)*

*“It’s wonderful, I loved that way of providing information.” (P15)*

*“Excellent tool, easy to access and quick to disseminate.” (P32)*

In class 3, one of the relevant words was "theme", as the podcast listeners emphasized that the topics covered in the episodes were important for knowledge, thus enabling proper care for children in everyday practice. For them, the episodes were an accessible and scientific way to solve daily doubts.

*“It added a lot, they cleared up my doubts about newborns, autism, various themes. More information for everyday life with my children.” (P16)*

*“Easy to access tool addressing important themes. It helps to be close to the discussed themes.” (P08)*

Access to the topics covered in the episodes was described as important and necessary, especially in times of pandemic and social distancing. Accordingly, the participants highlighted the ease of access and understanding, as well as the fact that the podcast is a way to bring scientific knowledge in a safe and qualified way, and is, therefore, a tool to combat fake news.

*“The issue of fake news also helps a lot, because I believed a lot of it, before listening to the podcasts, after the information, it cleared all my doubts.” (P16)*

*“It has eased access to the most important themes about something that is still much unknown, adding a lot to knowledge.” (P32)*

The terms associated in class 4 refer to the ease, practicality and simplicity of this educational health technology, since it has an audio format that is available on the platform, which makes it possible to listen to it at any time and place, easing the consumption of content along with daily tasks. The credibility of the project was also highlighted as an important point for the content to be consumed and put into practice.

*“The podcast is practical and can be listened to anywhere, while the person performs another activity.” (P11)*

*“I can listen anywhere, performing any daily task, making it the simplest and most effective way to acquire knowledge.” (P12)*

*“It allows content to be repeated as many times as necessary.” (P19).*

### Block 2 - The podcast as a tool to ease the understanding of child care, with emphasis on hand hygiene and the correct use of alcohol gel

In class 2, the word “understand” was the most relevant and the reading of the segments of the texts made it possible to understand that the podcast episodes were an enabling tool for the participants to better understand the care required by children in the face of Covid-19. The participants even highlighted that some ways of acting were rethought after listening to the episodes.

*“The way we should be careful about Covid-19 with children. We were so lost.” (P02)*

*“They were important for me to talk to the children or better understand what they were passing through, and it influenced my brother’s care.” (P37)*

The participants reported that the use of the project’s podcast during the pandemic period was of great value to them so that they could be better prepared to prevent and promote children’s health in a didactic and safe way.

*“Certainly, in health promotion and disease prevention.” (P07)*

*“The podcast helped me considerably to follow a safe path to promote my daughters’ health.” (P01)*

*“When I listened to the podcast about newborn stimulation during the pandemic, my nephew was not born yet; therefore, it was essential for me to be prepared to help him right after birth.” (P02)*

In class 5, the words “alcohol,” “hand,” “water” and “soap” appeared as some of the most recurrent and showed a strong association in this class. The reading of the text segments made it possible to understand that this topic addressed in one of the episodes entitled “The use of 70% alcohol during childhood” disseminated the appropriate knowledge for the correct use of alcohol, enabling its application in daily practice, in order to minimize the risks of using this substance at this stage of life, promoting health and providing prevention.

*“I wanted to sanitize my children's hands with alcohol all the time, without thinking about the risk of putting it within their reach and forgetting the basics, sanitizing with soap and water.” (P19)*

*“I stopped putting alcohol gel on the children’s hands, as they are not old enough to use it, and we started using only soap and water.” (P30)*

It was found that the topics covered in the podcast episodes are a way to prevent Covid-19-related problems and promote health when put into practice in everyday family life.

*“Currently, I have more information for everyday life with my children. Mainly, in terms of visiting newborns and preventing Covid-19.” (P16)*

*“It has helped me to learn things related to children in a simple way, I didn’t have to do long research.” (P35)*

## Discussion

The research results indicated that the podcast was recognized as an educational health technology that helped disseminate knowledge about child care. This technology proved to be enlightening, relevant, practical, accessible, and interesting. The topics addressed were important for expanding the listeners’ knowledge, emphasizing hand hygiene and the correct use of alcohol gel. The podcast’s audio format makes it easy to consume the content along with daily tasks.

Social media play an important role in information and communication technologies, and, as their engagement increases, they become an important resource for sharing guidance, easing the development of new health practices, and reaching a larger and more heterogeneous audience^16^. The podcast has been gaining ground, and as a social media, it brings science closer to society, being an ally in the disclosure of science to the population^17^, a premise that corroborates current findings.

The podcast was considered a relevant tool in providing guidance on child care, which is in line with a study carried out in Beirut, Lebanon. The study used Facebook to assess the impact of education on social media among health students and residents, confirming that social media is an important means of disseminating knowledge^18^.

The podcast as a social media was also pointed out as a practical and accessible health education technology, in agreement with a study carried out in Ceará, Brazil, about the use of podcasts for sexual and reproductive health education of adolescents, which highlighted the ease of access to this media^10^.

A study carried out in Israel, which used the same tool during the COVID-19 pandemic with medical students, also indicated ease of accessibility and integration into daily activities when using this technology^19^. Accordingly, the possibility of consuming the content while performing different tasks on a daily basis was pointed out as a factor of great importance among podcast listeners.

A Brazilian study with elderly people and caregivers using the same educational technology also pointed to podcasts as an accessible, comprehensive, and low-cost way to disseminate information, making them a relevant tool in health education^20^. Social media is an ally in distance education, being a strategy to combat misinformation and fake news, during and after the COVID-19 pandemic, which is why sharing truthful and easily accessible content with the population is essential^5^.

The findings made it possible to identify changes in the participants’ behavior after listening to the podcast, which corroborates the principles of health education in terms of seeking to change the population’s attitude towards newly learned knowledge. Accordingly, research carried out in Curitiba, Brazil, highlights health education as an instrument of social change, which aims to improve people’s quality of life, providing resources for them to become active participants in the health process^21^.

One should reinforce the importance of social media as a means of disseminating information to improve care practices aimed at the pediatric population. Consequently, a current strategy for dealing with the challenges and limitations of health professionals, including geographical limitations, could be the use of social media, including podcasts, which are a quick and economical way to make information more accessible and empower the population^22^.

Beyond the pandemic, it is crucial to recognize the importance of producing podcasts that address compelling topics related to children’s health. These podcasts play a key role in encouraging safe practices and providing appropriate health guidance for both families and the community. Providing care in the wrong way can damage children’s health, compromising their growth and development. It is, therefore, imperative to ensure access to correct information and promote health education as essential elements for childhood survival^23^.

The limitations of this study refer to the exclusion of digitally illiterate citizens, since an electronic device and internet access were required to listen to the podcast and participate in the survey, as well as the impossibility of helping the participants when they did not understand a question, which may have limited the answers. As there is no guarantee that the forms were answered exclusively by the study participants, this uncertainty represents another limitation. In addition, no studies were found on the use of podcasts for child care knowledge, making it difficult to deepen the discussion in this area. Thus, there is a need for further research with other methodological designs and in other settings, in order to expand knowledge about this topic.

## Conclusion

It was identified that the podcast is an educational health technology that eases disseminating knowledge about child care to the population in a free, practical, and accessible way and can, therefore, be applied to health education. The participants pointed out that the podcast had even resulted in more assertive care practices for children. They also praised the shared content and highlighted the relevance of the topics and the importance of this technology.

The podcast is an innovative and democratic health education technology whose format for reproduction and sharing eases its consumption in people’s daily lives, since it can be reproduced inside and outside the home environment, including while traveling. It is also an inclusive technology, as it allows people with visual impairments, who often find it difficult to achieve educational content suited to their needs on social media, to have free access to shared knowledge.

Podcast as a social media is capable of contributing to the prevention and promotion of the health of children and their families, including in the post-pandemic period, given the infinity of health topics that can be shared with the population, health professionals, and students since the use of social media is growing more and more. It is underlined that no scientific studies have been found about the use of podcasts to disseminate child care; therefore, this study is innovative from a digital health perspective.

## References

[1] Almeida EWS, Godoy S de, Silva ÍR, Dias OV, Marchi-Alves LM, Ventura CAA (2022). Salud digital y enfermería: herramienta de comunicación en la Estrategia Salud de la Familia. Acta paul enferm.

[2] Bezerra LS, Gibertoni D (2021). Social media during the covid-19 pandemic: behavior analysis of users during this period and the possibilities for the future. Rev Inter Tecnol.

[3] Ferentz L, Fonseca NM, Accioly NS, Garcias CM (2020). Hashtags related to covid-19 in Brazil: the usage during the beginning of the social isolation. Com Ciências Saúde.

[4] Silva MMS, Carvalho KG, Cavalcante IKS, Saraiva MJG, Lomeo RC, Vasconcelos PR (2020). Knowledge intersection in social media for health education in the covid-19 pandemic. Sanare Rev Pol Publ.

[5] Souza TDS, Ferreira FB, Bronze KM, Garcia RV, Rezende DF, Santos PR (2020). Social media and health education: combating fakes new a in the covid-19 pandemic. Enferm Foco.

[6] Cassiano AN, Silva CJD, Nogueira ILA, Elias TMN, Texeira E, Menezes RPM (2020). Validation of educational technologies: bibliometric study in nursing theses and dissertations. Rev Enferm Cent.-Oeste Min.

[7] Gonçalves MIA, Melo MEFA, Araujo TO, Antero MB (2021). Pandemic times: health education via social networks. Rev. Extensão UPE.

[8] Silva NVN, Pontes CM, Sousa NFC, Vasconcelos MGL (2019). Health technologies and their contributions to the promotion of breastfeeding: an integrative review of the literature. Ciênc Saúde Colet.

[9] Coradini NHK Borges AF, Dutra CEM (2020). Podcast educational technology in professional and technological education. Rev Ensino Interdiscip.

[10] Leite PL, Torres FAF, Pereira LM, Bezerra AM, Machado LDS, Silva MRFC (2022). Construction and validation of podcast for teen sexual and reproductive health education. Rev Latino-Am Enfermagem.

[11] WorldHealth Organization (2021). Globalstrategyondigitalhealth2020-2025.

[12] Borges JO, Góes FGB, Silva ACSS, Ávila FMVP, Goulart MCL, Nunes NGF Mendeley Data V1.

[13] Nascimento LCN, Souza TV, Oliveira ICS, Moraes JRMM, Aguiar RCB, Silva LF (2018). Theoretical saturation in qualitative research: an experience report in interview with schoolchildren. Rev Bras Enferm.

[14] Góes FGB, Santos AST, Campos BL, Silva ACSS, Silva LF, França LCM (2021). Use of IRAMUTEQ software in qualitative research: an experience report. RevEnferm UFSm.

[15] Minayo MCS (2012). Qualitative analysis: theory, steps and reliability. Ciênc Saúde Colet.

[16] Góes FGB, Nunes NGF, Borges JO, Souza AN, Soares IAA, Lucchese I (2023). Transmedia in pediatric nursing for guidance to family members in coping with covid-19: experience report. Rev Enferm UFSM.

[17] Casaes RS, Pereira BR, Marcellini PS, Pires DA, Ade GV, Matos YACS (2021). The use of scientific knowledge in the area of nutrition for podcast information and communication technology. Res Soc Dev.

[18] Atallah S, Mansour H, Dimassi H, Kabbara WK (2023). Impact of social media education on antimicrobial stewardship awareness among pharmacy, medical and nursing students and residents. Bmc Med Educ.

[19] Anteby R, Amiel I, Cordoba M, Axelsson CGS, Rosin D, Phitayakorn R (2021). Development and utilization of a medical student surgery podcast during covid-19. J Surg Res.

[20] Camacho CLF, Ferraz VHG, Silva JON, Barroso SA, Souza VMF (2022). Podcastasaneducationaltechnology for seniors and their caregivers: an experience report. Res Soc Dev.

[21] Conceição DS, Viana VSS, Batista AKR, Alcântara ASS, Eleres VM, Pinheiro WF (2020). Health education as an instrument for social change. Braz J Dev.

[22] Fernandes BMP, Santos CDMMLD, Coscarelli CT, Araujo JSD, Bezerra MADS, Gonçalves OC (2023). Use of social media associated with health promotion in dentistry. Population Medicine.

[23] Góes FGB, Ma Silva, Santos AST, Pontes BF, Lucchese I, Silva MT (2020). Postnatal care of newborns in the family context: an integrative review. Rev Bras Enferm.

